# Lactational performance of dairy cows fed ad libitum grass silages of different quality supplemented with concentrates high or low in locally sourced ingredients

**DOI:** 10.1016/j.vas.2026.100648

**Published:** 2026-04-06

**Authors:** Katrine Sømliøy Eikanger, Margrete Eknæs, Jon Kristian Sommerseth, Inger Johanne Karlengen, Ingunn Schei, Alemayehu Kidane

**Affiliations:** a Faculty of Biosciences, Norwegian University of Life Sciences, P.O. Box 5003, NO-1432 Ås, Norway; b TINE SA, BTB - NMBU P.O. Box 5003, NO-1432 ÅS, Norway; c Norgesfôr AS, Akershusstranda 27, 0150 Oslo, Norway

**Keywords:** Alternative protein, Alkaline barley, Non-protein nitrogen, Grass silage quality

## Abstract

Grass silage is central to Nordic dairy cow diets, but its variable quality – affected by weather and growing conditions - necessitates protein supplements. High-protein concentrates are used to optimize diets, but often include significant amounts of imported protein ingredients, negatively impacting sustainability and regional feed self-sufficiency. We evaluated possible interactive effects between concentrates containing a higher share of imported ingredients with soy (Control) as a protein source or higher share of locally sourced ingredients with alkalized barley (Alka) as a protein source, combined with two grass silage qualities differing in organic matter digestibility (OMD; HighOMD and LowOMD), on dry matter intake (DMI), digestibility, and milk production. The study involved 40 Norwegian Red (NRF) dairy cows in a continuous 2 × 2 factorial design experiment over 63 days (21-d adaptation, 42-d measurements). All cows received grass silage ad libitum, with concentrate allowance optimized based on Control, then replaced quantitatively by Alka for relevant groups.

Results revealed no interactions between concentrate type and grass silage quality. HighOMD silage improved grass silage DMI and nutrient digestibility (P < 0.001). A greater concentrate allowance for cows fed LowOMD silage compensated for the lower nutritional value of the basal diet in terms of milk production. Feed use efficiency was lower for cows fed LowOMD silage (P = 0.007). In conclusion, concentrates with a higher inclusion of locally produced ingredients and alkalized barley as a protein source can replace concentrates with a higher share of imported ingredients and soy protein when fed in combination with differing grass silage qualities.

## Highlights

• Similar performance with concentrates based on local or imported ingredients.
• Individually optimized concentrate levels compensate for silage quality.
• No interaction between concentrate type and grass silage quality.

## Introduction

Grass silage constitutes a significant share of dairy cow rations in the Nordic countries. Large variations in grass silage nutritive value put pressure on the protein quality of supplemental feeds for dairy cows. At-large, high-quality grass silage can supply sufficient amino acids absorbed in small intestines (AAT) for milk protein synthesis in high-yielding dairy cows (Randby et al., 2012). However, a decrease in digestible organic matter (OM) by a magnitude of 5.0 g/d with advancing growing days for primary spring growth (Kuoppala et al., 2008), varying due to latitude (Deinum et al., 1981) and unpredictable weather conditions for timely harvest, affects silage quality and yield putting further pressure on feed management. Therefore, diets for lactating dairy cows are optimized for protein and energy by including varying amounts and quality of concentrates. Currently, a large part of these concentrate ingredients, especially high-protein sources, is imported to Europe (Hristov et al., 2024) with consequences for the economy, sustainability, and environmental footprint of dairy production. As a result, there is a growing interest in covering the demand for protein sources in concentrates, based on local or regional resources. In the European Union, concrete measures taken to support the development of plant proteins to limit dependency, through Common Agricultural Policy, has increased areas for legume production (Agriculture & Rural Development, 2024). This is augmented with further investments in the field of protein crops and feeding systems under Horizon Europe and the European Innovation Partnership on Agriculture to make livestock production systems more sustainable, resilient, and circular (EU CAP, Network) even though some threats exist (Parisi et al., 2020). In the Nordics, production of such legume crops to cover the need for protein ingredients is even more challenging due to various reasons such as short growing season, crop failure and low yield (Belachew et al., 2026), and disease outbreak (Aamot et al., 2023; Ruokola & Vestberg, 1978). Thus, alternative approaches would help mitigate these problems. To this end, we (Eikanger et al., 2025) have demonstrated the feasibility of replacing concentrates formulated with high inclusion of imported ingredients, such as soy, corn and beet pulp, with concentrates high in locally produced cereal grain including alkalized barley as a non-protein nitrogen (NPN) source, combined with good-quality silage without compromising feed utilization and milk production with early lactation Norwegian Red (NRF) dairy cows.

However, the amount and quality of supplemental feeds are commonly chosen based on the level of milk yield (MY) and quality of basal diets. Effects of concentrate supplementation on MY are often positive (Lawrence et al., 2015; Roche et al., 2006, 2013), but the interactive effects of silage quality and concentrate feed type on total feed intake and milk production are not always as anticipated (Grøseth et al., 2024). In vitro rumen-simulated works involving various silage qualities and concentrate types suitable for dairy cow rations showed no interaction between fermentation parameters and estimated protein and energy values at practical feeding levels (Kidane et al., 2020; Muqier et al., 2023). The lack of interaction in the latter was alluded to the limitations of in vitro fermentation conditions.

Given the inconsistent or absent interactions reported between silage quality and concentrate type, we hypothesized that a concentrate feed with a higher share of locally sourced ingredients could effectively replace a commercial soy-based concentrate without negative effects on feed intake, diet digestibility, or milk yield. We further hypothesized that grass silage with higher OM digestibility (OMD) would support comparable milk production with reduced need for concentrate supplementation, compared to silage with lower OMD. Lastly, we expected that the effects of concentrate feed type would be independent of the grass silage quality. The outcomes were expected to have practical implications on sustainable milk production based on local feed resources with minimal recourse to concentrate feeds and imported ingredients, therein. Therefore, the objective of this study was to compare two roughly isonitrogenous and isoenergetic concentrate feeds formulated for commercial use, high or low in locally produced ingredients, combined with two grass silage qualities (High vs. Low OMD) on dry matter intake (DMI), nutrient digestibility, and milk production in NRF cows.

## Materials and methods

### Experimental design

A trial involving 40 NRF dairy cows was carried out at the Livestock Production Research Center of the Norwegian University of Life Sciences (Ås, Norway). The trial adhered by the laws and regulations set by the Norwegian Animal Research Authority, controlling in vivo animal experiments.

The trial was performed with a continuous 2 × 2 factorial design over 63 days, including 21 d adaptation and 42 d of measurements. The experimental treatments consisted of two pelleted concentrate feeds that differed in their inclusion of locally produced ingredients, with key distinctions in their protein- and carbohydrate sources (Table 1). These were: **Alka** – a concentrate feed high in locally sourced ingredients with alkalized barley (Alka grain 150 Barley, Strand Unikorn, Norway) as a source of NPN, and a higher overall inclusion of barley as the primary carbohydrate source; and **Control** – a commercial concentrate feed formulated for dairy cows fed grass silage-based diets, containing a lower proportion of locally produced ingredients, with soy as a protein source and imported carbohydrate ingredients such as corn and beet pulp. The two concentrate feeds were fed in combination with one of two grass silage qualities differing in OMD (early cut grass silage high in OMD = **HighOMD** vs. late cut grass silage low in OMD = **LowOMD**). Chemical composition of the feeds are provided in Table 2. Both concentrate feeds used in this study were selected based on performance from a previous screening trial (Eikanger et al., 2025), where Control and Alka corresponded to the Soya-F and Alka-F treatments, respectively. As described in Eikanger et al. (2025), the alkalized barley included in Alka was prepared by combining a compound mix including approximately equal parts of urea and full fat soy (Home n’ Dry, Strand Unikorn, Norway/ Dugdale Nutrition, UK) with whole barley grains in a 3:17 ratio. Water was added to the mix to achieve a target moisture content of approximately 18%, after which the mix was left covered under plastic for a minimum of two weeks. The two grass silages were both 1^st^ cuts, harvested from the same field with 11 days between cuttings, resulting in two silage qualities differing in OMD. The grass cuts were made from a timothy (*Phleum pratense*) based stand of a 1^st^ year ley seeded from a commercially available mix (Strand nr 15, Norgesfôr, Oslo, Norway) containing 60% timothy, 20% meadow fescue (*Lolium pratense*), 10% red clover (*Trifolium pratense*) and 10% perennial ryegrass (*Lolium perenne*). The wilted grass were preserved in round bales using the formic- and propionic acid based additive GrasAAT Plus (Addcon Nordic AS, Porsgrunn, Norway) applied at a rate of 4.5 L per ton.

**Table 1:** Ingredient composition (g/kg as is basis) of experimental concentrate feeds formulated with low (Control) or high (Alka) inclusion of locally sourced ingredients.

	Control	Alka
Alka grain 150 Barley^1^	–	200
Ground barley	369	532
Ground oats	120	70
Ground wheat	–	50
Wheat bran	111	–
Corn gluten meal	34	25
Ground corn	100	–
Beet pulp	80	–
Rapeseed expeller	–	30
Soybean product	99	–
Lipitec Bovi LM^2^	7	5
Molasses	50	50
Monocalcium phosphate	4	7
Magnesium oxide	4	7
Sodium sulfate	–	2
Vitamins and trace elements	23	22

1 Alkalized barley.

2 Rumen protected fat (Lipitec AS, Ringe, Denmark).

**Table 2:** Chemical composition and estimated energy and protein values of the experimental diet components.

	Concentrate		Silage quality	
	Control	Alka	HighOMD	LowOMD
DM%	90.0	89.7	22.8	24.5
Chemical composition, g/kg DM				
CP	175	170	157	126
Soluble CP^1^, g/kg CP	–	–	720	657
NDFom	195	154	532	599
ADFom	87.7	68.8	328	378
ADL	15.3	13.1	23.5	37.5
Starch	401	501	–	–
Ash	68.9	65.6	81.7	74.6
Crude fat	23.5	15.5	29.3	23.2
NFC	538	595	200	177
Estimated energy and protein values				
OMD, %	–	–	75.5	70.2
AAT_20_, g/kg DM	108	98	78	76
NEL_20_, MJ/kg DM	6.22	6.21	6.31	5.82
PBV_20_, g/kg DM	6	19	33	4
Silage fermentation quality				
Ammonia-N^1^, g/kg N	–	–	130	82
pH^1^	–	–	4.5	4.2

Control = concentrate with imported soy and carbohydrate ingredients; Alka = concentrate with high levels of locally sourced ingredients including alkalized barley; HighOMD = early cut grass silage with higher organic matter digestibility; LowOMD = late cut grass silage with lower organic matter digestibility;

DM = dry matter; CP = crude protein; NDFom = ash-corrected neutral detergent fiber; ADFom = ash-corrected acid detergent fiber; ADL = acid detergent lignin; NFC = non-fiber carbohydrates; OMD = organic matter digestibility estimated by near-infrared spectroscopy; AAT_20_ = standard feed value for amino acids absorbed in the small intestine at 20 kg of DMI/d; NEL_20_ = standard feed value for net energy lactation at 20 kg of DMI/d; PBV_20_ = standard feed value for protein balance in the rumen at 20 kg of DMI/d

1 Analysed by near-infrared spectroscopy.

### Animals and management

The cows were of mixed parity (n = 17 primiparous and n = 23 multiparous) in their early lactation with average (mean ± SD) days in milk (DIM) of 48 (± 18 days), MY of 24.1 (± 7.98 kg/d), and body weight (BW) of 565 (± 72 kg) at the start of the experiment. Cows were randomly allocated into one of the four treatments blocked by initial MY, parity, BW and DIM. The cows were housed in a free stall system with ad libitum access to grass silage and drinking water. Concentrates were provided in the milking robot, and automatic concentrate feeders (DeLaval, Tumba, Sweden).

The grass silages were prepared for feeding, three times a week, by chopping and mixing in a Siloking Duo 1814 mixing wagon (Kverneland, Bryne, Norway) for 20 min. Each batch of mixing was preserved using 4 L per ton wet feed of a propionic acid-based (32–42% Propionic acid, 12–16% Sodium Benzoate and 10–12% Sodium Propionate) preservative (GrasAAT FEED, Addcon Nordic AS, Porsgrunn, Norway).

Silage was offered in 40 feed troughs (20 for each silage quality; Biocontrol, Rakkestad, Norway) automatically recording intake and equipped with automatic Radio Frequency Identification readers giving the cows access only to troughs with the assigned silage quality. The troughs were calibrated once a week, while emptied, and cleaned out a minimum of every second day. The silage was manually controlled daily and refilled twice daily, ensuring ad libitum access. Concentrate allowance was calculated and optimized based on individual requirements at the start of adaptation with Control and the appropriate silage quality using TINE Optifôr (Nordic feed evaluation system; NorFor, 2011). The optimized daily concentrate allowance was 7.2 kg/d for primiparous and 10.2 kg/d for multiparous cows with HighOMD silage. When fed LowOMD silage, the optimized allowance increased to 8.6 kg/d for primiparous and 12.0 kg/d for multiparous cows, compensating for the differences in silage quality. Alka replaced Control on a weight-by-weight (w/w) basis. To meet the reduced energy requirement as cows progressed in lactation, the concentrate allowance was reduced by 5% every 21 days. The cows were milked in a milking robot a minimum of twice a day with an average number of 3.53 (± 0.208 n/d) visits. Access to the robot was restricted to every 6^th^ hour for each cow.

### Recording and sampling

Silage samples were taken three times a week for each treatment by picking silage from all troughs at different positions within the trough, with a portion analyzed for dry matter (DM) immediately, and another portion stored at −20 °C until further processing. The samples for each quality were pooled in three, corresponding to the early, mid, and late stages of the experiment and dried at 59 °C for 48 h before milling for the intended chemical analyses. The concentrates were sampled once a week from the automatic concentrate feeders, and the milking robot (DeLaval, Tumba, Sweden) and pooled into one sample per concentrate type, analyzed for DM to estimate concentrate DMI and stored at −20 °C until pooled as described for the silage samples into three samples for each concentrate corresponding with early, mid, and late stages of the experiment.

Total tract digestibility was estimated from fecal spot samples using acid insoluble ash (AIA) as described by Van Keulen and Young (1977). Feces samples were taken from all cows at three time points (0600, 1200, and 1900) over 72 h during the last week of the experiment and frozen at −20 °C until thawed and dried at 60 °C in preparation for analyses. Based on the variance estimates reported by Thonney et al. (1985), three fecal grab samples are sufficient to achieve reliable digestibility estimated for cattle fed forage-based diets. After drying, equal portions of the three samples per cow were pooled into one sample for further analysis.

Milk samples were taken for all visits in the robot over 24 h once a week, preserved with Bronopol tablets (2-Bromo-2-nitropane-1,3 diol, Broad Spectrum Microtabs II, Landteknikk, Økern, Norway), and stored at 4 °C. From these, three samples per cow were selected each week for analysis based on milk volume and interval between milkings (e.g., if a cow was milked four times over a 24-h period, with comparable yields for each milking, three samples spaced far apart from one another were selected). Body weight was automatically measured as the cows left the milking robot via a scale (Biocontroll, Rakkestad, Norway), which was controlled daily and calibrated weekly.

### Chemical analysis

Dried samples of silage, concentrate, and feces were milled to pass a 1.0 mm screen, using a cutting mill (SM 200, Retsch GmbH, Haan, Germany) and analyzed for DM, ash, AIA, crude protein (CP), neutral detergent fiber (NDF), acid detergent fiber (ADF), acid detergent lignin (ADL), and crude fat. A small portion of the concentrate samples were additionally milled to pass a 0.5 mm screen for starch analysis.

Dry matter and ash content were determined after drying at 103 °C overnight (ISO, 1999) and incineration at 550 °C (ISO, 2022), respectively. Acid insoluble ash concentration was determined using ISO method 5985 (ISO, 2002), and CP was calculated based on the total nitrogen concentration (N × 6.25) analyzed using AOAC method 2001.11 according to Thiex et al. (2002), with Kjeltec 24,002,460 Auto Sampler System (Foss Analytical, Hilleroed, Denmark). Neutral detergent fiber (NDF) content was determined according to Mertens (2002), using sodium sulfite and alpha amylase, with an Ankom^220^ fiber analyzer (ANKOM Technology, Fairport, NY, USA). Acid detergent fiber (ADF) was determined using AOAC Method 973.18 (AOAC, 2000), without acetone washing of samples. The NDF and ADF samples were corrected for residual ash content and are presented on organic matter (OM) basis as NDFom and ADFom, respectively. Acid detergent lignin (ADL) was extracted after ADF analysis with 72% sulfuric acid (H_2_SO_4_) for 3 h, before ashing to correct for inorganic materials. Concentrate samples were prepared for total starch analysis using AACC (2000) method 76–13.01; Megazyme Amyloglucosidase/alpha-Amylase assay protocol, hydrolyzing starch to glucose, before determining the concentration by colorimetric spectrometry using a RX DaytoNA^+^ spectrometer (Randox Laboratories Ltd., Crumlin, UK). Crude fat content was analyzed by accelerated solvent extraction using a Dionex ASE 200 Accelerated Solvent Extractor (Thermo Scientific, Waltham, USA) with 100 % petroleum ether at 100 °C for the silage samples, and 80% petroleum ether and 20% acetone at 125 °C for the concentrates.

Milk samples were analyzed at the TINE SA milk laboratory (Heimdal, Norway) for milk protein, fat, lactose, and urea using a Bentley FTS/FCM Combisystem mid-infrared milk analyzer (Bentley Instruments Inc., Chaska, Minnesota, USA).

### Calculations

Response variables calculated or estimated based on measured parameters are indicated here. Non-fiber carbohydrates (NFC) content in feeds were calculated as: 1000 – (CP + NDF + crude fat + ash) according to Sadeghi et al. (2025). Net energy intake as total intake (MJ/d) and per kg DMI (MJ/kg DMI) were calculated individually based on the net energy value for lactation (NEL) of the feeds.

The concentration of AIA in feeds (AIA_feed_) and feces (AIA_feces_) was used to estimate total tract dry matter digestibility (DMD_AIA_, %) according to Van Keulen and Young (1977) where: $DMD_{AIA}=\left(\frac{AIA_{feed}-AIA_{feces}}{AIA_{feces}}\right)\times \;100$.

Energy corrected MY (ECM) was calculated individually, based on MY (kg) and milk chemical composition (g/kg) according to Sjaunja (1990), as MY × [(38.30 × fat content + 24.20 × protein content + 16.54 × lactose content + 20.70)/3.140]. Nitrogen use efficiency (NUE) was calculated as N secreted in milk as a percentage of N intake, and feed use efficiency (FUE) was calculated as kg ECM produced per kg DM consumed.

The average daily BW change over the relatively short experimental period was estimated using a simple linear regression over the measurement days. Raw data records (n = 1680) from the electronic scale were subjected to outlier detection for each cow using the Interquartile Range Technique as described in Vinutha et al. (2018) and data points falling outside the lower or upper bounds (n = 243) were excluded. Average daily BW change for individual cow was then estimated as the slope coefficient of the fitted model of the form:

$$BW=\beta_{0}+\beta_{1}\times Day+e$$

where for the individual cow, BW= response variable, $\beta_{0}=$ intercept (i.e., the starting BW), $\beta_{1}=$ slope (i.e., the average daily BW gain), Day= predictor variable, and e= error term. The slope was then subjected to statistical analysis as described below.

### Statistical analysis

Statistical analysis was performed using R (R Core Team 2025 edition 4.4.3). A mixed model was used for all variables. For nutrient intake, MY, and milk composition all parameters were averaged per cow per week and the statistical model included individual cow as random effect. Concentrate type, silage quality, and the interaction between them were included as fixed effects in addition to experimental week, cow parity, and DIM. For apparent total tract digestibility, and BW change, ‘Week’ was excluded from the model as this data were not repeated. Pre-experimental covariate data was available for MY, ECM and milk composition, and covariates were therefore included in the model for these variables. An autoregressive (AR [1]) correlation structure was selected for variables with repeated measures based on Bayesian information criterion (BIC).

The model, without the effect of pre-experimental covariates, was as follows:

$$Y_{ijkl}=\mu +Concentrate_{i}+Silage_{j}+\;\left(Concentrate_{i}\times Silage_{j}\right)+Week_{k}+DIM_{l}+Parity_{m}\;+\;Cow_{n}+e_{ijklmn}$$

where $Y_{ijkl}$ = response variable (e.g., DMI); μ= overall mean; $Concentrate_{i}=$ fixed effect of concentrate type *i* (Control or Alka); $Silage_{j}=$ fixed effect of silage quality (HighOMD or LowOMD); $Concentrate\times Silage_{ij}=$ fixed interaction of concentrate type and silage quality; $Week_{k}=$ fixed effect of week during the measurement period (*k* = 1 to 6); $DIM_{l}=$ fixed effect of DIM; $Parity_{m}=$ fixed effect of parity (primiparous [1] or multiparous [≥2]); $Cow_{n}=$ random effect of individual cow and $e_{ijklmn}=$ residual error. Data are presented as estimated marginal means (EMM). Significance was declared at *P* < 0.05, and tendencies were noted for 0.05 ≤ *P* < 0.1.

## Results

### Feed intake and total tract digestibility of nutrients

The intake of DM from feeds and individual feed components (as shown in Table 3) did not reveal any interaction between concentrate type and grass silage quality. Additionally, there were no significant differences between the two concentrate types for intake parameters, apart from crude fat intake which was greater for Control compared to Alka (*P* < 0.001), and a tendency toward a higher intake of NFC for Alka compared to Control (*P* = 0.06). Grass silage quality influenced both silage and concentrate intake. Cows fed HighOMD silage had a 0.9 kg DM greater intake of silage (*P* = 0.05), and a 1.07 kg DM lower intake of concentrates (*P* < 0.001) compared with cows fed LowOMD silage. However, grass silage quality did not influence total DMI and OM intake. Intake of NDFom tended (*P* = 0.08) to be greater and the intake of ADFom and ADL was greater for cows fed LowOMD silage (*P* = 0.03 and *P* < 0.001, respectively). Lastly, cows fed HighOMD silage had a greater intake of CP and crude fat (*P* < 0.001) relative to cows fed LowOMD silage.

**Table 3:** Effect of concentrate feeds formulated with low (Control) or high (Alka) inclusion of locally sourced ingredients and two silage qualities differing in organic matter digestibility (high = HighOMD vs. low = LowOMD) on nutrient intake in NRF dairy cows.

	HighOMD		LowOMD			P-value^1^		
	Control	Alka	Control	Alka	SE	Con	Sil	Con × Sil
Intake, kg DM/d								
Silage	12.8	12.9	12.1	11.8	0.454	ns	0.05	ns
Concentrate	7.36	6.84	8.18	8.16	0.199	ns	<0.001	ns
Total DM	20.1	19.8	20.3	19.9	0.504	ns	ns	ns
OM	18.6	18.3	18.8	18.6	0.466	ns	ns	ns
NDFom	8.23	7.95	8.80	8.32	0.268	ns	0.08	ns
ADFom	4.84	4.72	5.27	5.02	0.167	ns	0.03	ns
ADL	0.42	0.39	0.58	0.55	0.016	ns	<0.001	ns
CP	3.29	3.19	2.96	2.88	0.073	ns	<0.001	ns
Crude fat	0.55	0.49	0.47	0.40	0.013	<0.001	<0.001	ns
NFC	6.34	6.47	6.39	6.79	0.135	0.06	ns	ns
Total NEL, MJ/d	126	124	121	119	3.0	ns	ns	ns
NEL, MJ/kg DMI	6.28	6.28	5.98	5.98	0.002	ns	<0.001	ns

Con = Concentrate; Sil = Silage; Con × Sil = Concentrate × Silage; DM = dry matter; OM = organic matter; NDFom = ash-corrected neutral detergent fiber; ADFom = ash-corrected acid detergent fiber; ADL = acid detergent lignin; CP = crude protein; NFC = non-fiber carbohydrate; NEL = net energy for lactation.

1 ns = not significant, tendencies (0.05 ≤ P < 1.0) are displayed.

Total intake of net energy for lactation (NEL) was not affected by interactions between or concentrate type and silage quality separately. When calculated as NEL intake per kg DMI, cows fed HighOMD silage had a significantly greater energy concentration intake than cows fed LowOMD silage (*P* < 0.001).

The total tract nutrient digestibility presented in Table 4 indicates no interaction by concentrate type and grass silage quality for the digestibility of the assessed nutrients. Similarly, no significant effects were found for concentrate type alone. However, the total tract digestibility of DM, OM, NFDom, ADFom and CP was influenced by grass silage quality (*P* < 0.001). Specifically, higher digestibility was observed for HighOMD silage for these parameters compared to LowOMD silage.

**Table 4:** Effect of concentrate feeds formulated with low (Control) or high (Alka) inclusion of locally sourced ingredients and two silage qualities differing in organic matter digestibility (high = HighOMD vs. low = LowOMD) on total tract apparent nutrient digestibility in NRF dairy cows.

	HighOMD		LowOMD			P-value^1^		
	Control	Alka	Control	Alka	SE	Con	Sil	Con × Sil
Digestibility, %								
DMD	82.9	84.4	75.5	75.1	1.02	ns	<0.001	ns
OMD	83.5	85.1	76.1	76.0	1.02	ns	<0.001	ns
NDFomD	77.0	79.4	66.1	65.1	1.77	ns	<0.001	ns
ADFomD	76.7	78.9	64.5	63.3	1.84	ns	<0.001	ns
CPD	81.2	82.1	74.9	72.5	1.02	ns	<0.001	ns

Con = Concentrate; Sil = Silage; Con × Sil = Concentrate × Silage; DMD = dry matter digestibility; OMD = organic matter digestibility; NDFomD = digestibility of neutral detergent fiber corrected for organic matter; CPD = crude protein digestibility.

1 ns = not significant (P ≥ 0.05).

### Milk yield, milk composition, and milk component yields

No interaction of concentrate type by grass silage quality were found for MY, ECM, any of the milk components, or milk urea nitrogen (MUN) (Table 5). Concentrate type did not influence milk yield and milk composition parameters apart from a significantly greater (*P* = 0.02) lactose content for Alka. Feeding HighOMD silage tended (*P* = 0.08) to increase milk production relative to the LowOMD silage, with a 1.7 kg/d mean difference in MY. This was reduced to a numerical difference of 0.4 kg/d when calculated as ECM. Grass silage quality did not influence fat and lactose composition in milk. However, a strong tendency toward greater milk protein content (*P* = 0.05) was observed for HighOMD silage. Milk fat yield did not differ significantly between silage qualities, but milk protein yield was significantly higher (*P* = 0.01), and lactose yield tended to be higher (*P* = 0.07) for cows fed HighOMD silage. Similarly, MUN content was not affected by either concentrate type or silage quality.

**Table 5:** Effect of concentrate feeds formulated with low (Control) or high (Alka) inclusion of locally sourced ingredients and two silage qualities differing in organic matter digestibility (high = HighOMD vs. low = LowOMD) on milk yield and milk composition in NRF dairy cows.

	HighOMD		LowOMD			P-value^1^		
	Control	Alka	Control	Alka	SE	Con	Sil	Con × Sil
Yield, kg/d								
MY	27.7	27.4	25.8	25.9	0.93	ns	0.08	ns
ECM	29.9	29.6	29.1	29.7	0.50	ns	ns	ns
Milk composition, %								
Fat	4.24	4.23	4.22	4.34	0.100	ns	ns	ns
Protein	3.67	3.56	3.52	3.49	0.046	ns	0.05	ns
Lactose	4.81	4.83	4.78	4.84	0.019	0.02	ns	ns
Milk component yield, kg/d								
Fat	1.16	1.16	1.13	1.12	0.045	ns	ns	ns
Protein	0.99	0.98	0.91	0.91	0.031	ns	0.01	ns
Lactose	1.33	1.32	1.23	1.25	0.046	ns	0.07	ns
Other variables								
MUN, mg/dL	12.9	12.2	12.7	12.8	0.26	ns	ns	ns
NUE, %	30.4	30.7	29.9	31.0	0.95	ns	ns	ns
FUE, kg ECM/kg DMI	1.57	1.59	1.34	1.47	0.052	ns	0.007	ns
Body weight change, kg/d	0.60	0.45	0.35	0.43	0.142	ns	ns	ns

Con = Concentrate; Sil = Silage; Con .. Sil = Concentrate .. Silage; MY = milk yield; ECM = energy corrected milk, calculated according to Sjaunja (1990); MUN = milk urea nitrogen; NUE = nitrogen use efficiency, nitrogen secreted in milk as percentage of nitrogen intake; FUE = feed use efficiency.

1 ns = not significant, tendencies (0.05 ≤ P < 1.0) are displayed.

### Bodyweight change, and feed and nitrogen use efficiency

Daily BW change, apparent nitrogen use efficiency (NUE), and feed use efficiency (FUE) were not influenced by interactions of concentrate types and grass silage qualities or concentrate type alone (Table 5). Grass silage quality did not influence daily BW change. However, the calculated FUE averaged about 9.5% higher for cows fed HighOMD silage compared to those fed LowOMD silage, with a significant difference (*P* = 0.007).

## Discussion

### Chemical composition of feeds

The primary objective of this study was to evaluate production responses of dairy cows fed grass silage differing in quality combined with pelleted concentrate feeds formulated either with a high inclusion of locally sourced ingredients and protein provided through NPN, or with a lower inclusion of locally sourced ingredients and with soy as a protein ingredient under practical feeding conditions. The two concentrates were formulated to provide approximately isoenergetic and isonitrogenous diets while meeting macro- and micronutrient requirements for lactating dairy cows.

Overall, Control represented a typical commercial concentrate used in grass silage-based feeding systems in Norway (Alvarez et al., 2022; Weiby et al., 2025). It consisted of roughly 50% barley and other locally grown cereal grains, supplemented with commonly imported protein- and carbohydrate ingredients such as soybean meal, maize and beet pulp. In contrast, Alka contained approximately 85% locally grown cereal grains. Alkalized barley was included at a level so that ammonia-N originating from the alkalization process substituted soy protein on a 1:1 basis, supplying around 25% of the total CP from concentrate.

The estimated metabolizable CP supply, expressed as amino acids reaching the small intestine (AAT) at 20 kg DMI (AAT_20_; NorFor, 2011), of Control was 10 g/kg DM higher than that of Alka. In contrast, the potential supply of rumen degradable protein relative to the available NEL (protein balance in rumen; PBV) at 20 kg DMI (PBV_20_; NorFor, 2011) indicated a greater ratio between rumen available N and rumen available energy for Alka than for Control.

For the two grass silage qualities, the 11 days between harvests reduced the concentration of digestible OM by 5.3 g/d, which is consistent with the decline estimated by Randby et al. (2012), and as recently reported by Alvarez et al. (2022). As a consequence of the greater content of structural carbohydrates and a lower CP content, OM digestibility and the estimated NEL_20_ was lower for LowOMD silage. Based on the grass silages characteristics, the optimized concentrate allowance was therefore higher for cows receiving LowOMD silage compared to HighOMD silage. Crude protein solubility for grass silages decreases with increasing plant maturity, as more protein is bound to less digestible structural carbohydrates (Rinne et al., 1997). Thus, HighOMD silage was calculated to provide a greater balance of rumen soluble N to the available energy, giving a 29 g/kg DM difference of PBV_20_ between the two silage qualities.

### Feed intake, digestibility and production response

In the current study, changing the ingredient composition of concentrates, by excluding commonly imported concentrate ingredients and increasing the use of readily available local ingredients, did not affect intake or digestibility of the basal grass silage. The absence of interaction between concentrate formulation and grass silage quality suggest that alkalization of high-starch ingredients may allow for greater inclusion of locally sourced barley across grass silages of differing quality. Our results align with studies showing that silage quality has a larger impact on dairy cow performance than concentrate carbohydrate source (Keady & Mayne, 2001). Sadeghi et al. (2025) previously reported that manipulating the macronutrient composition of a total mixed ration by increased forage NDF and fat, negatively affected intake and digestibility compared to a high-starch low-fiber and low-fat diet without interaction of protein source when slow-release urea partially replaced soy. A lack of influence on grass silage DMI and OMD with increased inclusion of barley and NPN replacing imported carbohydrate and protein by use of concentrates similar to Control and Alka were also previously observed with protein sufficient diets in Eikanger et al. (2025), and in the study by Kidane et al. (2022) where untreated barley quantitatively replaced soy in pelleted concentrates.

Grass silage DMI is positively correlated with silage DM digestibility (Huhtanen et al., 2007), and a greater intake of HighOMD grass silage was therefore expected. This was also demonstrated under similar conditions by Randby et al. (2012) and in a recent study in the same herd, where Alvarez et al. (2022) reported a higher silage DMI when cows were offered grass silage with higher OMD (78.8%) compared to grass silage with lower OMD (67.6%). The same study (Alvarez et al., 2022) also demonstrated the need for a greater concentrate allowance when feeding grass silage with lower OMD to cover energy and protein requirements and sustain comparable milk production.

Milk urea nitrogen concentration is related to the dietary CP content, particularly the intake of PBV (Monteils et al., 2002; Nousiainen et al., 2004). Nousiainen et al. (2004) estimated a MUN level of 11.7 mg/dL with a protein and energy balanced diet (i.e., PBV = 0). A positive PBV of 10–40 g/kg DM for dairy cows producing >20 kg ECM/d is generally recommended by NorFor (2011). The observed MUN levels, given the theoretical PBV-values for the feeds included here, are considered reasonable and consistent with previously reported values for early-lactation NRF dairy cows fed grass silage-based diets (Randby et al., 2012).

Contrary to Sadeghi et al. (2025), who found an interaction for MUN between protein source and diet composition, we found no interaction or individual effect of concentrate formulation and silage quality. Sadeghi et al. (2025) observed this interaction when partially replacing soy with slow-release urea, combined with either a high-starch low-fiber low-fat or low-starch high-fiber high-fat basal diet. In their study, MUN was greater with the low-starch high-fiber high-fat diet and further increased with slow-release urea partially replacing soy, without statistical differences in CP intake or MY. Sadeghi et al. (2025) speculated that the combination of low-starch and slow-release urea might result in a less efficient protein metabolism.

Because concentrate allowance in the present study was adjusted to meet individual requirements for each designated silage quality, with the aim of achieving comparable ECM production, silage quality did not affect ECM production. There was however a tendency towards a greater milk yield for cows fed HighOMD silage. The absence of statistical difference in MY, despite a 1.7 kg discrepancy, can be attributed to the high variability in MY among individual cows.

### Feed utilization

Overall, the experimental diets provided sufficient protein and energy for the targeted ECM production and to support additional live weight gain requirements. When protein and energy requirements are met, the source of protein becomes secondary, though still important (Brito & Broderick, 2007; Kidane et al., 2022). Concentrate formulation did not affect milk fat and protein composition, in agreement with Haig et al. (2002), who found that milk fat and protein composition were not affected when increasing NPN (% of CP) from 5.9 to 11.7% by replacing soy with urea in balanced diets. Broderick and Reynal (2009) also demonstrated that increasing replacement (0, 1.2, 2.4 or 3.7%) of rumen degradable protein from soy with urea only influenced milk fat and protein yield due to lower MY.

The alkalization of high-starch feed ingredients is expected to enhance the synchronization of energy and N availability for microbial protein synthesis, thereby increasing the supply of metabolizable protein in the intestines. In an in vitro study by Muqier et al. (2023), where metabolizable protein was estimated as the utilizable crude protein (uCP) content of feeds, an alkalized barley-based diet produced uCP values higher than that of a soy-based concentrate feed. This was observed both when combined with grass silages of contrasting qualities and in its pure form. Higher supply of metabolizable protein is expected to increase both milk yield and FUE as reported recently by Benchaar et al. (2023). However, because milk and milk protein yield respond to increased metabolizable protein (i.e., AAT) supply in a curvilinear manner, any advantage from increased microbial CP synthesis is likely reduced when the diets already provide sufficient protein coverage (Nousiainen et al., 2004). Thus, the lack of difference in milk yield and FUE between Control and Alka diets are notable, given that Control had a higher estimated potential for delivering AAT (i.e., 10 g/kg DM). This may suggest that microbial CP compensated for this difference for cows fed Alka. However, the overall dietary CP concentration was likely too high to distinguish potential reasons with certainty.

Our recently published study (Eikanger et al., 2025), did nonetheless, demonstrated an increase in FUE for cows fed concentrate including alkalized barley, compared with a concentrate based on the same ingredients as Alka, but with added feed grade urea as the source of NPN. In comparison, Rauch et al. (2025) observed no difference in FUE when replacing feed grade urea with a rumen resistant coated urea. The same study also found that fully substituting soy with added feed grade urea or coated urea resulted in a reduction of FUE by about 12%, attributed to a decrease in DMI (Rauch et al., 2025). Whereas Libera et al. (2021) reported a 9% increase in ECM when NPN from an alkalized grain mixture replaced approximately 20 g soy in a maize and grass silage-based TMR. The increased ECM production was attributed to more favorable rumen conditions. Although Libera et al. (2021) did not report FUE, the experimental groups were provided with the same feed allowance, established as 95% of the ad libitum voluntary intake prior to the experimental period. Therefore, the calculated difference in FUE would be equivalent to the difference in ECM. These findings contradict the similar FUE found for concentrates including soy or alkalized barley in both Eikanger et al. (2025) and the present study. The control diet provided by Libera et al. (2021) might, however, have limited microbial CP synthesis compared to the diet including alkalized grain, as indicated by a lower dietary CP concentration and lower milk urea, as well as a lower rumen fluid microbial count and milk protein yield, explaining the contrasting results found here.

In the present study, cows fed HighOMD silage had a greater daily intake of CP as well as NEL per kg DMI, thereby increasing energy available for milk protein production (Brun-Lafleur et al., 2010) without affecting NUE. Due to the tendency towards lower MY with LowOMD silage, the protein yield was also lower. The higher content of forage NDF in the LowOMD silage likely stimulated increased acetate production (Van Houtert, 1993), leading to similar milk fat yields (Urrutia & Harvatine, 2017) for both silage qualities and explaining the lack of significance in ECM production in the present study.

To compensate for the readily available energy lost during ensiling and inefficient capture of grass silage N in rumen, high-starch high-protein concentrates are included in the diet to cover the dairy cow’s energy demands. In the present study, diets were formulated to meet the cow’s protein requirements, which might have masked a potential effect of nutrient synchrony provided by Alka (Hall & Huntington, 2008).

## Conclusions

Results from the present study did not indicate any interactions between concentrate feed type and silage quality (i.e., high- or low-OMD silages), apart from minor numerical differences. Overall, silage quality affected DMI, diet digestibility and milk yield independent of the concentrate formulation. Thus, alkaline grain-based concentrate feeds with increased content of local ingredients can replace soy-based concentrates with lower inclusion of local ingredients in dairy cow diets at high and low grass silage OMD levels, corresponding to the range of grass silage qualities tested here. The observed milk and milk component yields with the HighOMD grass silage, in combination with lower DMI from concentrate feeds, suggested that high-quality grass silage can sustain milk production with less reliance on concentrates, benefiting cost and sustainability.

## Funding statement

The Norwegian Research Council funded this study through the project “Alkaline Grain Technology: Increased Norwegian Ingredients in Cattle Diets and Effects on Milk Production, Animal Health and GHG Emissions” (Project #302341 – AlkaNor), with support from local (Norgesfôr AS, Strand Unikorn AS, and TINE SA) and international (Dugdale Nutrition, UK) industrial partners.

## CRediT authorship contribution statement

**Katrine Sømliøy Eikanger:** Writing – review & editing, Writing – original draft, Visualization, Validation, Resources, Methodology, Investigation, Formal analysis, Data curation, Conceptualization. **Margrete Eknæs:** Writing – review & editing, Writing – original draft, Validation, Supervision, Resources, Methodology, Investigation, Conceptualization. **Jon Kristian Sommerseth:** Writing – review & editing, Resources, Methodology, Conceptualization. **Inger Johanne Karlengen:** Writing – review & editing, Resources, Methodology, Conceptualization. **Ingunn Schei:** Writing – review & editing, Resources, Methodology, Conceptualization. **Alemayehu Kidane:** Writing – review & editing, Writing – original draft, Visualization, Validation, Supervision, Resources, Project administration, Methodology, Investigation, Funding acquisition, Formal analysis, Data curation, Conceptualization.

## Declaration of competing interest

The authors declare the following financial interests/personal relationships which may be considered as potential competing interests: I.J. Karlengen works for the company providing the concentrate feeds for the experiment reported. The remaining authors declare that they have no known competing financial interests or personal relationships that could have appeared to influence the work reported in this paper.

## Data Availability

Data from the reported experiment is available upon request to the corresponding author.
